# The PICO project: aquatic exercise for knee osteoarthritis in overweight and obese individuals

**DOI:** 10.1186/1471-2474-14-320

**Published:** 2013-11-13

**Authors:** Flávia Yázigi, Margarida Espanha, Filomena Vieira, Stephen P Messier, Cristina Monteiro, Antonio P Veloso

**Affiliations:** 1 Department of Sports and Health, Univ de Lisboa, Fac Motricidade Humana, CIPER, LBMF, P-1499-002 Lisbon, Portugal; 2 Department of Health and Exercise Science, Wake Forest University, Winston-Salem, NC, USA

**Keywords:** Aquatic exercise, Knee osteoarthritis, Exercise, Pain, Obesity

## Abstract

**Background:**

Aquatic exercise is recommended by the Osteoarthritis Research Society (OARSI), by the American College of Rheumatology (ACR) and by the European League Against Rheumatism (EULAR) as a nonpharmacological method of controlling the knee osteoarthritis (KOA) symptoms. Moreover, given that weight loss results in a reduction of the load that is exerted upon the knee during daily activities, obesity is also considered to be a modifiable risk factor for the development and or exacerbation of KOA. The implementation of an exercise based weight loss program may, however, itself be limited by the symptoms of KOA. The aquatic program against osteoarthritis (termed “PICO” in Portuguese) prioritizes the control of symptoms and the recovery of functionality, with an attendant increase in the patient’s physical activity level and, consequently, metabolic rate. Our laboratory is assessing the effectiveness of 3 months of PICO on the symptoms of KOA, on physical function, on quality of life and on gait. In addition, PICO shall examine the effects of said exercise intervention on inflammatory biomarkers, psychological health, life style and body composition.

**Methods/Design:**

The trial is a prospective, single-blinded, randomized controlled trial, and involves 50 overweight and obese adults (BMI = 28–43.5 kg/m^2^; age 40–65 yrs) with radiographic KOA. The participants are randomly allocated into either an educational attention (control) group or an aquatic (exercise program) group. This paper describes the experimental protocol that is used in the PICO project.

**Discussion:**

The PICO program shall provide insight into the effectiveness of an aquatic exercise program in the control of KOA symptoms and in the improvement of the quality of life. As such, they are likely to prove a useful reference to health professionals who intend to implement any kind of therapeutic intervention based around aquatic exercise.

**Trial registration:**

NCT01832545.

## Background

Although rheumatic diseases (RD) have low death rates, they are one of the primary causes of compromised quality of life and absenteeism from work, with attendant economic and social consequences [1]. In Portugal, RD are responsible for 40 to 60% of situations of prolonged physical incapacity in daily activities, for 43% of cases of absenteeism from work, and for 35 to 41% of early retirements due to illness [2].

Osteoarthritis (OA) is the most prevalent rheumatic disease and represents a great risk to the quality of life of the individual, given the consequent loss of autonomy that can be precipitated by its effect on lower extremity based activities (such as walking up and down stairs, climbing and squatting) [1,3,4]. Although the causes of OA are not completely understood, it is thought to be a complex, adaptive response of the joints to biomechanical, genetic and environmental stresses [5]. Recent studies demonstrated that that low-grade inflammation plays a pathophysiological role in OA. The severity, the symptoms, the impairment in physical function and the progression of OA may also be partly mediated by the extent of chronic inflammation in OA patients [6,7].

The knee is the most commonly affected weight bearing joint, being considered the 4^th^ and 8^th^ main cause of disability in women and men, respectively [8]. KOA is more common in the medial tibiofemoral compartment than in other sites of the knee, probably due to a higher frequency of varus malalignment [9]. Inflammation and joint loading are commonly believed to cause or to exacerbate the disease process [10]. Obesity, prior knee injury, physical activity levels, physical strength and the extent of alignment/misalignment of body segments are the most often cited potential risk factors for KOA in the academic literature. The latter risk factor seems to have more importance in the radiographic progression than in the incidence of KOA [11-14].

Knee Osteoarthritis (KOA) is highly prevalent in obese individuals [15]. The International Association for the study of Obesity (IASO)/International Obesity Taskforce (IOTF) analysis (2010) estimated that approximately 1.0 billion adults are currently overweight and a further 475 million are obese, worldwide [16]. Obese individuals have higher concentrations of the inflammatory markers (such as TNF-α and leptin) that are predominantly secreted by adipose tissue and can induce the production of IL-6 and C-reactive protein (CRP) [17]. The pathogenesis of obesity is characterized by hypothalamic inflammation and subsequent central resistance to leptin. High leptin concentrations then compromise the reduction of food intake and increase in energy expenditure. In addition, leptin increases the synthesis of a stimulator of osteophyte formation, TNF-β [18]. The resulting low- grade inflammation plays a pathophysiological role in OA, because it can affect muscle function and lower the individual’s pain threshold. It can also affect chondrocyte homeostasis and cause degenerative changes in cartilage [6,10,19].

Besides its effect on the individual’s quality of life, KOA uses up considerable health care resources. The consequences of KOA make it a public health problem in many countries [1,20-22]. Pain is the symptom that markedly affects quality of life in KOA patients. Gait tests are an important measure of mobility and KOA patients may adapt their gait and adjust body alignment to reduce pain. However, the latter adaptations may increase the loading on the joint and result in increased disease progression. In addition, the pain that is concomitant with KOA causes irritability, sleeplessness, depression and other physical and psychological changes that may aggravate the disease and incur both a general loss of functionality and a drop or maintenance in physical activity levels to below the recommended levels [23]. The main consequences of inactivity are weight gain and the obesity installation [24]. The combination of obesity and KOA create a vicious cycle of pain, loss of functionality, and disease progression. To interrupt this cycle, KOA symptoms should be reduced. Physical activity and weight loss can make an important contribution.

Current therapy most often focuses on pain relief, using mainly analgesics and nonsteroidal anti-inflammatory medications that have only a modest functional benefit and do not slow disease progression, whilst causing serious cardiovascular and gastrointestinal side effects [25,26]. The recommendations of the Osteoarthritis Research Society International (OARSI), the American College of Rheumatology (ACR), and the European League Against Rheumatism (EULAR) also include exercise as an important treatment [27-29]. Aerobic, aquatic, and resistance training exercise are recommended [27].

### Exercise program and Knee OA

Appropriate exercise can provide an improvement in symptoms and reduce pain, preventing OA-associated disabilities and increasing quality of life. In addition, there seems to be a positive effect of exercise on the chondroprotective anti-inflammatory cytokine response [30], mediated by IL-10 and IL-4. The weight loss and body composition improvements that can induced by exercise, reduce the thigh fat depots and may have a positive effect on the secretion of pro-inflammatory cytokines, lowering IL-1 and leptin levels in individuals with KOA [10].

The literature indicates that an exercise intervention for KOA should be broadly based and include aerobic training, lower limb-strengthening, gait training, flexibility, stability and posture training, weight reduction, patient education and psychosocial intervention [27,31-34].

Aquatic exercise is at this moment one of the main non pharmacological interventions that is suggested by the OARSI, ACR and EULAR guidelines as a means of controlling the symptoms and to prevent or slow down the progression of KOA [26,27,35]. Various studies have demonstrated that controlled aquatic exercise classes can be effective for KOA symptom control and to improve function [36-43]. In addition, a person in pain has difficulty with weight bearing exercise. Aquatic exercise allows aerobic exercise to be accomplished with less load on the joints [44].

Several studies have examined the effects of exercise in water (hydrotherapy, aquatic exercise) on the symptoms of knee arthritis [36-40,44-51]. However, said studies are inconsistent as regards the quality and quantity of exercise that was performed. There is a lack of definition of what is involved in hydrotherapy and what is performed in aquatic exercise classes. Aquatic Exercise (AE) is an exercise modality which can be defined as a group of exercises performed in the water, mainly in the vertical position, with or without music, with or without equipment added and in shallow or deep water. Its main characteristics are the utilisation of hydrostatic pressure and hydrodynamics to work on the aerobic and neuromuscular system. AE has been used for rehabilitation (hydrotherapy) [52-59], for athletic training [60-64] or for general fitness activity [65-71].

Aerobic training is very important for pain control and to improve functionality in individuals with KOA [27]. According to the guidelines of the Aquatic Exercise Association (AEA) [72], aerobic aquatic exercise classes with fitness goals, should be performed in water between 28-30°C so as not to compromising the endocrine responses. Water temperatures above 32°C are recommended for passive work, relaxation techniques or for individuals with low movement levels, but are not advisable temperature for aerobic or strength based exercise [64]. In cases of patients with low aquatic abilities, the Arthritis Foundation Guidelines suggest a superior limit range of water temperature of 31°C [73].

The PICO program is an overall body fitness and mind based intervention through aquatic exercise involving an educational component that has been specifically created for individuals with KOA. Two recent studies regarding the aquatic exercise in KOA should be referred to [38,39], as they have a controlled methodology and were performed according to the AEA guidelines [72].

#### *Study aim*

To design an aquatic exercise program specific to knee osteoarthritis, with the goal of management of OA symptoms and the improvement of physical fitness. The PICO program is based around the first step for weight loss interventions in individuals with KOA being the control of pain and other symptoms. When KOA symptoms are controlled, patients learn that it is possible to live with the disease which in turn motivates lifestyle changes. The increase of physical activity due to lifestyle changes may then cause improvement in body composition. In this way 6 hypotheses are formulated: H1. 3 months of aquatic exercise will improve KOA symptoms (pain and stiffness) and physical function in obese individuals with KOA; H2. The gait parameters (gait speed, gait cycle, ground reaction forces and knee forces moments) of obese individuals with KOA can be improved by aquatic exercise; H3. Beyond fitness component and the exercises skills, aquatic exercise group classes can work as an educational component and to promote lifestyle changes. H4: The aquatic exercise program can improve mental status and quality of life. H5: The aquatic exercise program can cause a weight reduction or body composition change. H6: 3 months of aquatic exercise can have a positive effect on selected inflammatory biomarkers of KOA.

## Design/Methods

### Study design

**Figure 1:**
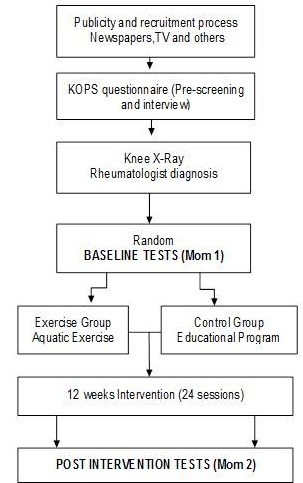
PICO flowchart.

PICO is a single-blinded, randomized controlled trial with 3 months duration. Participants will be randomly assigned to one of two groups: aquatic exercise (AE) and control group (CG). Figure 1 provides a flowchart of PICO design. Researchers and personnel responsible for data collection will be blinded four the group classes. The study protocol was approved by the Ethical Committee of the Faculty of Human Kinetics, Technical University of Lisbon. All participants will be informed about the procedures and potential risks and an informed consent will be obtained from them.

#### *Sample*

Considering the calculation of the sample size, the studies of Messier (2009) [74] and Wang (2007) [44] were used as a reference, because they had a sample with similar characteristics and studied similar outcomes. Both showed that a reduction of symptoms in patients with OA of the knee led to a significant improvement (α < 0.05) of the primary outcome measure of self-reported physical function. To find an analogous improvement of self-reported physical function of approximately 25% between the baseline and final measurements in patients with KOA, the minimum number of patients that are required is 20 for the main outcomes. The latter number is based on a power (1-B) of 0.80 and a significance level of 5% (two-sided). When a dropout rate of 20% is taken into account, at least 25 participants must be involved at study onset.

#### *Inclusion criteria*

The study sample will consist of 56 community-dwelling adults from the Lisbon area with: (1) age between 40 and 65 years; (2) 28.0 ≤ BMI ≤ 45 kg/m^2^; (3) unilateral or bilateral KOA diagnosed by knee pain and grade I-III radiographic tibiofemoral OA or tibiofemoral plus patellofemoral OA (4) a sedentary lifestyle, defined as not participating in a program that incorporates more than 30 minutes per week of formal exercise, within the 6 months leading up to the study; (5) being independently mobile; and (6) literate.

#### *Exclusion criteria*

The exclusion criteria are: subjects with skin diseases, with unstable medical conditions and or who have undergone hip or knee replacement, or knee surgery within the 6 months prior to the study; and participants who had any type of knee injections within the past 3 months.

#### *Trial conduct*

#### *Recruitment*

The recruitment and selection process will be done according to the aforesaid eligibility criteria. To avoid convenience sampling different documents will be created for advertising and publicizing the PICO project. Social networks, television interviews, newspapers and the collaboration of entities/companies will be the main channels for PICO announcements.

All eligible individuals who contact the study secretariat will go through the same selection process, namely: a telephone based pre–selection stage followed by a face to face interview and completion of the screening questionnaire which will supply information about demographics and symptoms, signs and risk factors for KOA occurrence. Volunteers who, after completing the questionnaire, fulfill the ACR criteria for clinical diagnosis [75] will receive a request for an x-ray examination. The exams will be referred to a rheumatologist who will make the final diagnosis according to ACR radiological criteria. In the case of confirmation of the KOA diagnosis, the subject will be invited to a further interview the purpose of which is detailed explanation of the project, obtaining his/her informed consent and familiarization with both the locale and the equipment which shall be used in the tests.

### Intervention programs

#### *Aquatic exercise (AE)*

The aquatic program is based on the guidelines of OARSI and EULAR for KOA management [28,29], on the Aquatic Exercise Association Guidelines (AEA) [72], on the Arthritis Foundation Aquatics Program instructor’s manual [73], on the ACSM’s Guidelines for Exercise Prescription [76] and on analysis of the protocols of previous studies [36,38,39,44,77,78].

The strategies in the PICO proposal to produce a high quality aquatic exercise intervention are structured according to AEA guidelines [72] and include overload assessment and managements, impact level control (level 1, 2 or 3), exercise cadence control and adequate music according to exercise goals. In addition the use of different and pre-defined cool-down sessions each week, a strong motivational component and pain assessment should make the difference in this protocol. Educational themes shall also be addressed during the classes. One educational theme per week shall be reinforced, such as: *What did you have for breakfast? Let’s try, during this week, to improve the quality of our breakfast. Next class I will check what you ate; remember that you should assess yourself about knee pain, let’s try to learn how to live with it.*

Another aspect that will be considered is the level of aquatic ability of each participant. Exercises like underwater breathing and flotation will be used in small doses to reduce fear of water and to ensure that participants are comfortable moving around the pool.

The aquatic program will be organized into 24 sessions distributed over 12 consecutive weeks, with a frequency of twice a week. The duration of each session will be 60 minutes, being that 10 minutes are for patient reception, blood pressure control, pain registry etc., and the effective time inside the water is 45 minutes. The indoor pool works with an air temperature around 27 ± 1°C and water temperature is controlled at 30.5 ± 0.5°C.

Table 1 describes the aquatic exercise class format for the first four weeks. Workouts are organized in order to have a progressive overload every week. Water is the main instrument to create resistance and only in the last week, according to the level of progression of the participants and whether their self-reported pain is controlled, will additional equipment be added to increase the water resistance and consequently, the exercise overload. The Omni - Perceived Exertion Scale for Resistance Exercise (OMNI-RES) and for aerobic target zone (0–10 Borg Scale) will be used according guidelines for exercise intensity management [76].

**Table 1:** The aquatic exercise protocol design for the first 4 weeks of the PICO program

**Music BPM**	**Week1-5 (128 bpm)**	**Week 1**	**Week 2**	**Week 3**	**Week 4**
**Warm up **(5-8′)	**Duration**	8′	8′	8′	8′
	**Patterns**	Walking combined with static and gentle movements of main joints. Use hands to keep thermal comfort	Walking combined with static and gentle movements of main joints	Walking combined with static and gentle movements of main joints	Walking combined with static and gentle movements of main joints
**Aquatic adaptation**		Vertical balance	Vertical balance	Submersion	Submersion
		Travellings	Travellings	Underwater breathing	Underwater breathing
**Aerobic** (15-30′)	**Duration**	3x5min	4x5′	4x5′	(1x10′) + 5′ + 5′
	**Exercise pattern**	Walking patterns with variation of upper limb patterns	Walking patterns with variation of upper limb patterns	Walking patterns with variation of upper limb patterns	10′ walking patterns + (2x5′) de basic aquatic patterns (Jumping jack, Ski, Leg Curl and Kicks)
	**Intensity (%HRmax and RPE scale)**	57%-67% RPE 4-6	57%-67% RPE 4-6	57%-67% RPE 4-6	64-74% RPE 5-6
	**Impact level**	1	1	1	1
**Strength**	**Repetitions**	2x8	1 x16	2x16	2x16^t1^ + 1x8^tt^
	**Cadence**	t1 = water tempo	t1 = water tempo	t1 = water tempo	t1 = water tempo tt = land tempo
	**Sets interval**	Active 1-2′	Active 1-2′	Active 1-2′	Active 1-2′
	**Equipment added**	No	No	No	No
	**Intensity (Omni-RES)**	6-7	6-7	6-7	6-7
**Cool down **(5-6′)		Static stretching on the wall	Static stretching (center)	Dynamic/static stretching (center)	Dance + Dynamic/static

RPE- Rate of perceived exertion scale.

The exercise instructors will use behavioral techniques: 1) to encourage social contact between participants; 2) to promote frequent contact during all intervention phases; 3) to define clear behavioral goals and allow feedback on achievements; 4) to help participants to self-monitor their pain and exercise intensity to complete activity; 5) to establish personal commitment to the project through the exercise leader.

#### *Educational program: control group (CG)*

The control group will not be enrolled in any exercise program but will participate in the educational program “PESO comunitário”. This program is based on appropriate clinical guidelines and on validated behavior change principles [79]. Implemented by an intervention team with expertise gained from current scientific research in weight control determinants, this program, as is PICO, is free of charge for all interested adults who wish to manage their weight and health. It has operated since 2005 with the objective of preventing obesity or reducing excess weight as well as some of the risk factors for adult obesity, via through a change to steady healthy habits, attitudes and behaviors. PESO lasts 3 months and comprises 12 sessions of one and a half hour, on a weekly basis.

### Measurements

#### *Screening measurements*

The **Knee Osteoarthritis Pre-Screening questionnaire** (KOPS) was validated by the authors and considered useful for this purpose (article in submission process). KOPS addresses physical function, activity level, co-morbid diseases, KOA risk factors and symptoms, height and weight (to determine BMI) as well as caregiver status. The demographic data that are collected include data on occupation, income, and educational level. The medical form is used to determine co-morbidities and to analyze any self-reported information on medications. The overall KOPS score has acceptable to good reliability with a Cronbach’s Alpha of 0.747 and satisfactory internal consistency with an Intraclass Coefficient (ICC) for average measures of 0.646. The results for test-retest of one-week interval for each component ranged from 0.895-0.992 for ICC and from 0.894 to 0.979 for Cronbach’s Alpha.

### Osteoarthritis diagnosis and severity classification

To confirm OA and classify its severity the same X-ray protocol will be used for all subjects. Bilateral, anterior-posterior, weight-bearing knee radiographs will be used to identify OA in the tibiofemoral joint, and sunrise views to identify OA in the patellofemoral compartment. Severity of tibiofemoral OA will be measured using the K-L grading scale [80].

#### *Outcomes, measures and instruments*

All tests will be performed by all subjects at both baseline and 3 month later, at the end of the exercise intervention, using the same protocols and evaluated by the PICO team member(s). Tests list can be checked in the Table 2. The tests and questionnaires will be distributed over two nonconsecutive days, taking the fatigue levels of the subject and the need to avoid overload into account. Therefore the knee strength test and the Six Minute Walking Test will be conducted on different days.

**Table 2:** Tests list

**ASSESSMENT (TEST)**	**Screening**	**Baseline**	**3 months**
**Recruitment**			
Interview	x		
KOPS	x		x
Knee X-ray (lateral and antero-posterior)	x		
**KOA symptoms and quality of life (Self-reported questionnaires)**			
Pain assessment (Brief pain inventory)		x	x
Knee osteoarthritis associated quality of life (KOOS)		x	x
MOS (SF-12v2)		x	x
Depression assessment (Beck depression inventory)		x	x
Weight and lifestyle inventory		x	x
**Physical function and gait**			
Aerobic capacity (6MWT)		x	x
Strength		x	x
Functional (FRSTS)		x	x
Knee (BIODEX dynamometer)		x	x
Hand (grip dynamometer)		x	x
Flexibility (CRS and BS tests)		x	x
Gait Analysis (Kinematics and kinetics)		x	x
**Morphology and body composition**			
Morphological measures (Anthropometry)		x	x
Body composition (DXA scan)		x	x
**Life style**			
Physical activity level (IPAQ)		x	x
**Inflammation biomarkers**			
Cytokines (blood drawn)		x	x

The main outcomes will be KOA symptoms (pain and stiffness) and quality of life, physical function (aerobic capacity, strength, and flexibility) and gait (kinetics and kinematics). The secondary outcomes are body composition, morphology, physical activity level, and inflammatory biomarkers. During the study, all participants will be allowed to maintain their usual medication, including analgesics and NSAIDs. A detailed record of medication will be done at baseline and 3 month post-intervention testing.

### Knee OA symptoms and health quality of life

**Brief Pain Inventory (BPI)** – short version. A consensus panel, the Initiative on Methods, Measurement, and Pain Assessment in Clinical Trials (IMMPACT) recommended the inclusion of the short version of Brief Pain Inventory (BPI) in all trials that intend to assess chronic pain [81]. It is a widely used, reliable, valid instrument that assesses pain history, location, intensity and its interference with daily activities in individuals with osteoarthritis [82]. The Portuguese version was validated and recent studies have provided strong support for its reliability and validity [83,84].

**Numerical Rating Scale (NRS).** The subjects shall learn to self-assess their knee intensity pain via the 0–10 point NRS. The NRS should be used to control pain intensity before and after the aquatic exercise class and whenever is necessary.

**Knee Injury and Osteoarthritis Outcome Score (KOOS).** This questionnaire includes 5 dimensions to measure KOA specific health-related quality of life (QOL), knee pain (Pain), other disease-specific symptoms (Other Symptoms), activities of daily living (ADL), sport/recreation function (Sport/Rec). A score in each of the five dimensions is calculated as the sum of the items included and then converted according to a 0–100 scale, with 0 representing extreme knee problems and 100 representing no knee problems. The KOOS is validated for patients with knee injury or with knee OA and is a reliable and responsive self-administered instrument for short-termfollow-up [85]. The Portuguese validation has acceptable reliability with Cronbach’s alpha coefficients between 0.77 and 0.95, and ICC ranging from 0.82 to 0.94 for the KOOS subscales [86].

Medical Outcomes Study **(MOS) Short-Form Health Survey (SF-12v2).** SF-12 v2 consists of a subset of 12 items to assess health status, organized into two domains: Physical (Physical Component Summary, PCS) and Psychological (Mental Component Summary, MCS) that make up the original SF-36 [87]. It has been shown that the SF-12 correlates highly with SF-36 in both obese and non-obese patients [88]. The Portuguese version of SF-12 has satisfactory reliability and validity [89,90].

**Beck Depression Inventory (BDI-II).** This instrument, developed by Beck and colleagues [91] has 21 items to rate the severity of depression according to the clinical definition. It is one of the most popular and widely used instruments for assessing the severity of depressive symptomatology. The Portuguese version [92] shows a good internal consistency, a factor structure very similar to the original version [91,93], and an adequate convergent validity.

### Physical function

**Six Minutes Walking Test (6MWT).** The distance (d) and gait speed (v) of the 6MWT is used to assess the aerobic capacity and the walking ability. It will be performed individually and according to the *American Thoracic Society protocol* (ATS) [94], in a controlled indoor environment 46 meters in length and rectangular in shape. The 6MWT is highly reproducible in obese individuals (r = 0.926; 95% CI 0.816-0.972, P < 0.001) [95], and it has been used in studies with KOA [75,96-100].

**Chair Sit and Reach test (CSR).** The CSR test is a safe and socially acceptable alternative to traditional floor sit-and-reach tests as a reasonably accurate and stable measure of hamstring flexibility [101]. The subjects shall be allowed three attempts for each limb and the best of these scores shall be recorded to the nearest centimeter.

**The Back Scratch Test (BS).** The BSS is a measure of overall shoulder range of motion which involves measuring the distance between (or overlap of) the middle fingers behind the back with a ruler [102]. After a familiarization trial, this test is assessed twice, alternately with both hands, and the best value of each registered.

**Five-Repetition Sit-To-Stand Test (FRSTST).** This is a widely used measure of functional strength. ICC values for it demonstrate from good to high test-retest reliability for adults and subjects with osteoarthritis [100,103,104].

### Strength

**Knee Strength.** A dynamometer Biodex System III (Biodex Medical Inc., Shirley, NY, U.S.A.) will be used for isokinetic assessment of knee strength (flexor and extensor muscles) and isometric knee strength, bilaterally. The first leg to be tested shall be the less affected leg or, in doubtful cases, the dominant leg. Gravity correction to torque is calculated at 45 degrees (0 = straight leg). The range of motion for testing is pre-determined from 20° to 80° for all subjects. The exclusion of extreme ranges of knee motion was established due to the fact that they are known to be painful and frequently non-executable by these patients, namely full extension due to quadriceps weakness Similar procedures, according to the dynamometer used, have been adopted in other surveys involving the same population [105-110].

Maximal Isokinetic Strength is measured on a concentric/concentric mode, at angular velocity of 60°/s. Among the low velocities’ group, the 60°/s have been used in other studies and should provide better stability for this population [111]. The subjects should perform one set with minimum overload for habituation and 2 sets of 3 repetitions with a 120-second rest between sets. The subjects should be oriented to perform the test by exerting maximum pressure on the isokinetic arm through the entire range of movement. During the test vigorous verbal encouragement shall be given to each subject. The set with higher extension peak torque together with the lowest coefficient of variation will be chosen for analysis.

The angle of best torque of the knee extension obtained from each subject in the isokinetic test should be chosen for the isometric test. Maximal knee extension isometric test will be applied in one set of 3 repetitions during 5 seconds with 30 seconds relaxation interval between repetitions. The best of the three force-time curve will be chosen according to the higher peak.

Prior to measurement, the subject shall be are seated on the dynamometer with knee and hip joints at 90°. Crossover shoulder, belly and knee straps, as well as a lap belt will be used to restrain the subjects movement. The lever arm pad is strapped around the lower leg, 2 centimeters above the lateral malleolus of the fibula. The axis of rotation of the dynamometer should be aligned with knee joint’s axis of rotation. Joint warm-up will be done by gentle free movements of flexion and extension of the knee.

**The Handgrip Strength Test (HST).** This test evaluates maximal isometric force of the muscles of the hand and forearm. Although the sample of this study will not have hand OA, this test has been used in obese individuals as an indicator of total body strength and functionality [112,113]. The adopted protocol for this project is the same that was used for Portuguese adults in the national observatory [114]. Prior to the test, the grip dynamometer should be adjusted to the size of the hand of each subject. Subject will be standing with arms along the body without contact with the trunk and elbow slightly bent at 200°. Testing is first done by dominant hand followed by the non-dominant one. The force must be performed during the expiratory phase and valsalva maneuver should be avoided. After three trials, if the difference between each value is within 3 kg, the test is considered completed. If a bigger difference is observed, then the test will be repeated after sufficient rest time. The best repetition will be chosen for further analysis.

### Morphology and body composition

**Body composition.** Body mass index will be calculated as mass (measured in kilograms by a standard calibrated scale) divided by height squared (measured in meters). A DXA scanner (QDR 4500A, fan-beam densitometer, software version 8.21; Hologic, Waltham, USA) will be used to measure whole body lean mass (LM), fat mass (FM) and bone mineral content (BMC). DXA measures the attenuation of X-rays pulsed between 70 and 140 kV synchronously with the line frequency for each pixel of the scanned image. According to the protocol described by the manufacturer, a step phantom with six fields of acrylic and aluminum of varying thickness and known absorptive properties shall be scanned alongside each subject to serve as an external standard for the analysis of different tissue components. The same technician shall position the subjects, perform the scans and execute the analysis using the standard analysis protocol. Based on test-retest using 10 subjects, the coefficient of variation (CV) in PICO staff for FM and FFM was 2.6 and 1.5% respectively, and the total error of measurement (TEM) and the CV were 0.02 kg and 1.7% respectively.

**Anthropometry.** The anthropometric measurements that shall be collected by this study shall include height, body mass, perimeters (waist, hip, middle thigh, patella shank and foot breadth. The aforesaid measures shall be collected by an ISAK accredited anthropometrist using procedures established by ISAK [115] except in the case of patellar circumference, foot diameter and abdominal sagittal diameter, which shall be obtained according to previously validated procedures [116-118]. The intra-observer technical error for circumferences and diameters measures in the pilot study ranged from 0.2 -0.4.

### Gait analysis

The gait protocol used on the present study was adapted from IDEA study [77], taking into account our laboratory specific equipment.

Data collection and analysis: Motion capture of the gait will be collected with 10 cameras Qualisys (Oqus-300) operating at a frame rate of 200Hz. Forty six reflective markers should be placed in predefined anatomical protuberances and used for the reconstruction of lower limb segments using Visual 3D software (C-Motion, Inc., Germantown, MD). To reduce noise, the motion data is filtered, using a low pass Butterworth filter, with a cutoff frequency of 15Hz [119]. Ground reaction force will be collected with two force platforms (Kistler AG, Winterthur, Switzerland) and AMTI (Advanced Mechanical Technology, Inc, Watertown, MA), synchronized with the motion capture system.

The test will be performed in a fifteen meters walkway, six successful trials are collected from each participant and three should be chosen for subsequent analysis. A trial consists of starting on the platform approximately 2 meters behind the initial timer and walking past the first beam of light to activate the timer. As the participant walks and passes the second beam of light the timer will stop and speed will be recorded. The participant will turn around and wait for orientations to perform similar trial in the opposite direction.

In general, KOA subjects walk at a slower speed and cadence, with a prolonged stance phase, presenting a static and dynamic varus alignment, showing smaller flexion and greater knee adduction moment (KAM) [12,34,120,121]. The following kinematic and kinetic outcomes should be analyzed from the gait test:

#### *Gait speed*

Freely chosen speed is slower in individuals with Knee OA being correlated with high ground reaction forces during heel strike [77];

#### *Gait cycle characteristics*

Swing and stance phase duration, stride frequency, stride length, knee and hip ROM and angular velocity;

#### *Ground reaction forces*

The vertical, anteroposterior and mediolateral force components will be recorded with a force platform. Computer software will be used to calculate duration, amplitude and impulse of the reaction forces;

#### *Knee adduction moment (KAM)*

Knee adduction moment (KAM) is considered a valid parameter to infer the level of mechanical loading [122]. Healthy subjects show substantially higher abduction moments than OA subjects and the external adductor moment has been linked to the development and progression of medial compartment OA in association with the varus alignment installation by increasing the compressive forces across the knee [123]. In addition, there is a significant inverse association between the peak of knee adduction moment during late stance and the amount of knee pain which may represent a compensatory mechanism to reduce medial tibiofemoral joint load in the setting of knee pain [124];

#### *Adduction angular impulse (AddImp)*

The integral of the frontal plane external joint moments (adduction and abduction) over time during the stance phase, providing a functional measure of gait rather than normalized KAM. This measure has been useful to distinguish loads in different OA severity [125,126];

#### *Knee extension moment*

Individuals with knee pain and weak muscles, as a protective mechanism, seem to avoid the quadriceps muscle recruitment during load acceptance in stance phase, showing a reduced knee peak extension moment;

#### Hip extensor moment

OA subjects walking at similar speeds of healthy one’s maintain their walking speed by increasing hip range of motion and its speed. Greater hip extensor moments may indeed help to maintain walking speed, but this does not appear to be the case with the hip flexor and ankle plantar flexor moments, which were substantially lower;

#### *Hip external abduction moment*

Individuals with knee OA seems to have a higher involvement of hip adductor muscles to compensate a weak quadriceps and hip abductors [12,127].

Since this is a longitudinal study, walking speed may change and should be measured at each testing moment (baseline and after intervention). One successful trial is defined as the one in which the participant’s entire foot is placed on the surface area of the force platform while walking within ± 3.5% of the freely chosen speed.

The freely chosen speed should be assessed in the biomechanical test day before the placement of markers, in so far as each subject shall walk barefoot in the walkway until a stabilization of walking speed is observed. Usually the latter stabilization occurs after crossing of the walkway 5–6 times. The speed is monitored using an infrared photocell control system (Model E3F2-R4B4-M, OMRON) set with 7.3 m apart at the hip level, interfaced with a processor specifically built to record the time and calculate the speed as a function of the distance.

### Lifestyle

#### *International physical activity questionnaire (IPAQ)*

The short form of IPAQ was chosen because it is easy to apply. Despite its liability having been verified in many countries and with different populations [128-130], studies have indicated that the IPAQ-SF typically overestimates physical activity [131]. However, this instrument will be used for controlling the amount of physical activity along the study, and not for any classification of physical activity level. All participants shall receive a previous explanation about how to complete the questionnaire, and their answers will be confirmed during the interview. Data will be processed according to IPAQ guidelines [132].

#### *The weight and lifestyle inventory (WALI)*

The WALI is designed to obtain demographics information, weight and dieting histories, eating and exercise habits, and relationships with family and friends [133]. The Portuguese version (IPEV) and the process by which it was translated is published in a national book [134]. The PICO project will use only the sections G, K and Q of the Portuguese version (IPEV) for controlling alcohol and tobacco consumption, dietary patterns and clinical historic.

### Inflammatory biomarkers

Our primary measures are IL-6 and IL-10. These cytokines are consistently implicated in OA pathogenesis and showed significant improvement with weight loss in ADAPT. We will also measure hsCRP as an overall marker of chronic inflammation and TNFα and TNFα soluble receptor 1 (sTNFR1) because they are also implicated in OA pathogenesis. Leptin, an adipokine that increases synthesis of TGFβ, a known stimulator of osteophyte formation, will also be measured.

For assessing IL-6, IL-10, hsCRP, TNFα, sTNFαR1 and leptin, venous blood samples (approximately 10 ml per visit) will be collected into dry tubes and EDTA tubes by standard procedures in the morning after a minimum of 4 h fast without any type of exercise.

Blood will be centrifuged at 1500 x g for 10 min to separate serum from the cloth in the dry tubes and plasma from red blood cells in the EDTA tubes. Serum and plasma will be frozen at 80°C for posterior analysis by ELISA using commercially available kits.

### Statistical analysis

Descriptive statistics, including frequencies for categorical variables and means with standard deviations (SD) for continuous variables with normal distribution and median for skewed distributions will be used to describe subject’s characteristics. Normality will be tested using the Kolmogorov-Smirnov test. For continuous outcome measures, differences in mean change (baseline minus post-intervention) will be compared between groups using analysis of covariance adjusted for baseline values of the outcome. Comparisons between groups (CG and AEG) at baseline and post intervention will be conducted by Independent-sample t-tests or Mann–Whitney U test if equal variance is not assumed. Changes within group will be analyzed by paired Student’s t tests or by the Wilcoxon Signed Rank test when normality is not assumed. A mixed model analyses of covariance (ANCOVAs) will be conducted for interaction analyses adjusted for BMI with two within-participant factors of 6MWT (baseline) and 6MWT (post-intervention) and between two groups (CG and AE). Statistical analysis will be performed using IBM SPSS Statistics 20.0 and MedCalc Statistical Software (MedCalc Software, Mariakerke, Belgium). Multiple Linear regression analyses will be performed to understand the potential covariates that could improve the explanation of the variability of outcomes (6MWT, strength and KOOS dimensions). Statistical significance will be set at P *<* 0.05 (2- tailed) for all analyses. Effect sizes will be calculated for all measures with an effect size of 0.2 considered small, 0.5 medium and 0.8 large.

## Discussion

The need to improve non-pharmacological intervention for patients with KOA is obvious, and aquatic exercise is an option for obese patients since it minimizes joint load. Although water exercise is frequently encouraged, relatively little research has been conducted in this area as compared to land-based exercise.

There are several strengths to the design of this study. The first one, is the detailed exercise prescription protocol concerning dosage (frequency, duration and intensity of the exercise), the fulfillment of overload and individuality principles of training (e.g., gradual increase of the number of sets and repetitions on strength training), and the control of exercise intensity during the sessions using rating perception effort scales (e.g., Borg RPE and Omni scales).

Secondly, the pool where the program will be delivered is easily accessed by train or bus. This aspect is crucial as the access to appropriate facilities and patient motivation to undertake water exercise, might be a barrier to adherence. Additionally, the fact of our sample being adult and not elderly should facilitate the displacement to our facilities.

Thirdly, the exercise program will be delivered by high qualified aquatic exercise instructors, all of whom shall be graduates in Sports Science at the Faculty of Human Kinetics who have specialized in exercise and health and fitness group skills. The exercise program will be delivered similarly to both classes regarding exercises, overloads and leading strategies according to the predefined plan. Four instructors will be organized in pairs, and each pair will take care of one aquatic exercise group during the entire program.

With the exception of the knee radiographs for OA diagnosis, all measures will be obtained in the same facility at the baseline and immediately after the end of the program. One barrier at each visit/measuring point will be the capacity to asses all participants in a short interval. Due to the specificity of the outcomes will be necessary to manage the schedule among four different labs. Therefore, to support the project, staff team includes one secretary responsible for administrative work, four technicians conducting the dynamometer tests, gait analysis, body composition and anthropometry assessments and one professional for collecting blood samples. Each technician received the responsibility to be specialized in two test’s group plus questionnaires and, to avoid inter-rater error, the same technician should lead its application in both assessments, baseline and post-exercise intervention. Participant adherence to exercise is one of the main challenges, therefore to avoid drop outs, motivational cues, intra-group social interaction, frequent telephone calls and the quality of instructors are the main strategies chosen to contribute to adherence rates. Besides, since our sample will include adults that may still be working which will create some difficulties in the definition of class schedules, one extra class, every 15 days, will be provided to enhance high compliance, and to allow that all participants might attend to 24 sessions during the 3 month period over which the study shall run.

One possible constraint to the success of the aquatic exercise programs would be the level of water skills of each participant. Although it is not necessary to know how to swim, the autonomy and the ability to apply power against the water are essential to get benefits from this type of activity, especially when performing strengthening exercises. Therefore some aquatic adaptation exercises will be introduced in the class format (Table 1).

Our study is based on the premise that individuals with KOA need a wide exercise intervention, adapted not only to the affected joint but to the health status of the patient, working the whole body and the mind simultaneously. In addition, we expect that aquatic exercise, beyond the improvement of KOA symptoms, can increase the symmetry between forces in the lower-limb joints (adduction/abduction and flexion/extension moments at knee and hip joints), so improving gait.

The format of the sessions, the study duration and the weekly frequency of exercise classes are organized in such a way as to make sure that this proposal is executable, not only for this project but also for future implementations by communities or in private pools. The sample size of this protocol are reduced because we consider that, before implementing the program in the general community, for public health, the exercise protocol should be validated by a very controlled process.

## Conclusions

This study is a Randomized Controlled Trial (RCT) using aquatic exercise specially designed with a very controlled methodology. It is expected that the results will enable evidence-based recommendations for the treatment of patients with KOA through aquatic-exercise. Future studies would aim to reproduce the protocol contained herein and implement it in a larger sample and in different communities. The findings of PICO’s aquatic program for KOA should inform the development of an effective and reproducible exercise protocol, available for use by any professional with aquatic exercise and exercise and health related knowledge.

## Competing interests

The authors declare that they have no competing interests.

## Authors’ contributions

FY conceived the study, participated in its design and coordination, shall carry out the biomechanical gait tests, the intervention team coordination and drafted the manuscript. ME participated in its design, coordinates staff, and improved both the manuscript improvement and data management. SM participated in the protocol development, and in training staff for biomechanics tests. FV participated in the design of the body composition and anthropometry based protocols and their coordination. CM coordinates and carries out the biomarker analyses. AV participated in the study design and manuscript improvement. All authors read and made comments on previous drafts of the manuscript, and approved the final manuscript.

## Pre-publication history

The pre-publication history for this paper can be accessed here:

http://www.biomedcentral.com/1471-2474/14/320/prepub
